# Evolutionary dynamics and geographical dispersal of *Borrelia lusitaniae*

**DOI:** 10.3389/fmicb.2024.1330914

**Published:** 2024-02-06

**Authors:** Valentina Cirkovic, Gorana Veinovic, Daliborka Stankovic, Darko Mihaljica, Ratko Sukara, Snezana Tomanovic

**Affiliations:** ^1^Group for Medical Entomology, Centre of Excellence for Food- and Vector-Borne Zoonoses, Institute for Medical Research, National Institute of Republic of Serbia, University of Belgrade, Belgrade, Serbia; ^2^Natural History Museum in Belgrade, Belgrade, Serbia

**Keywords:** *Borrelia lusitaniae*, phylogeography, phylodynamic, migratory birds, *Ixodes ricinus*, lizards

## Abstract

**Background:**

*Borrelia lusitaniae* is a species within the complex *Borrelia burgdorferi* sensu lato, associated with lizards as reservoirs and *Ixodes ricinus* as its main vector. *Borrelia lusitaniae* is predominantly distributed in Central and Southeast Europe, and in countries of the Mediterranean basin, such as Portugal, Morocco, Tunisia, and Italy where this spirochete appears to infect vector ticks more frequently than other genospecies. Evolution of this zoonotic tick-borne microparasite is shaped by different environmental factors. Comprehensive phylogenetic analysis may give insight into how *B. lusitaniae* spreads to new geographic locations.

**Aim:**

We applied Bayesian statistical methods to *B. lusitaniae* multilocus sequence typing (MLST) data to study the migration routes of *B. lusitaniae* and its potential for further spread.

**Results:**

The discrete phylogeographic analysis placed origins of *B. lusitaniae* in Southeast Europe and identified at least two introductions of *B*. *lusitaniae* from Europe to North Africa. Estimated effective reproductive potential (Re), as a key indicator for a pathogen spread, suggested potential for further spread.

**Conclusion:**

The results of this study can provide beneficial information about the potential for further spread of *B. lusitaniae* in Europe and North Africa and estimation of necessity for the development of strategies to monitor and control Lyme borreliosis.

## Introduction

Lyme borreliosis (LB), a tick-borne infectious disease caused by spirochetes of the *Borrelia burgdorferi* sensu lato (s.l.) complex, is the most widespread vector-borne disease in the temperate climates of the Northern Hemisphere (Stanek and Strle, 2018). The skin lesion-erythema migrans is the most frequent manifestation of LB but *Borrelia* can disseminate from the skin and affect the nervous system, joints, heart and/or eyes (Stanek and Strle, 2018). Currently, 21 *Borrelia* species, distributed across North America, Europe, Asia, and Chile, are classified in the *B. burgdorferi* s.l. complex (Ivanova et al., 2014; Stanek and Strle, 2018). In general, the distribution of each *Borrelia* species is associated with specific host species. Accordingly, *Borrelia afzelii* is mainly associated with rodents, *Borrelia garinii* and *Borrelia valaisiana* with birds, *B. lusitaniae* with lizards and *Borrelia spielmanii* with dor-mice (Richter and Matuschka, 2006; van Duijvendijk et al., 2015). Five *Borrelia* species are directly associated with human LB in Europe [*B. afzelii, B. garinii, Borrelia burgdorferi* sensu stricto (s.s.), *B. spielmani*, and *Borrelia bavariensis*], and additional three species: *B. lusitaniae, B. valaisiana*, and *Borrelia bissettii* have been detected or isolated from human samples, but with unclear human pathogenicity (Collares-Pereira et al., 2004; Rudenko et al., 2009).

*Borrelia lusitaniae* was first isolated from *Ixodes ricinus* tick in Portugal in 1993 (Núncio et al., 1993) and described as a species in 1997 (Le Fleche et al., 1997). The first case of *B.lusitaniae* infection in humans was registered in Portugal and correlated with clinical symptoms of LB, followed by the isolation of the strain from the skin of the patient with chronic skin lesions, characterized by two ill-defined erythematous macules associated with a local diffuse infiltration of the subcutaneous tissues (Collares-Pereira et al., 2004). Additionally, isolation of *B. lusitaniae* was also reported from a 13-year-old female child with vasculitis syndrome (de Carvalho et al., 2008).

This *Borrelia* species is maintained in a natural transmission cycle involving tick vectors (*I. ricinus*) and lizards as main reservoirs (Amore et al., 2007; Norte et al., 2015). Several species of the family *Lacertidae*, including *Psammodromus algirus, Podarcis* spp, *Teira dugesii* as well as *Lacerta* spp. were proposed as potential reservoir hosts for *B. lusitaniae* (Norte et al., 2021). The geographic distribution of *B. lusitaniae* is mostly limited to Central and Southeast Europe, and in countries of the Mediterranean basin, such as Portugal, Morocco, Tunisia, and Italy (Younsi et al., 2001; Sarih et al., 2003; Baptista et al., 2004; Bertolotti et al., 2006). It is also found in Slovakia, Moldova, Bulgaria, and Ukraine but with lower prevalence (Postic et al., 1997; Gern et al., 1999; Christova et al., 2001; Weiner et al., 2018). Interestingly, the studies conducted in recent years showed an unusual dominance of *B. lusitaniae* over other *Borrelia* species in Serbia (Milutinović et al., 2008; Potkonjak et al., 2016; Cakić et al., 2019).

Previously published results regarding the spread of *B. lusitaniae* indicated its focal distribution determined by the necessity of the simultaneous presence of *I. ricinus* as the main vector and lizards as the main reservoir. The question that remains to be answered is how *B. lusitaniae* spreads to new geographical areas. To date, only few studies traced the origin and dispersal of *Borrelia* species (*B. burgdorferi s.s*. and *B. garinii*,) in America using Bayesian statistics (Walter et al., 2017; Rudenko et al., 2023). In this study, we examined the geographic origin and migration routes of *B. lusitaniae* including its origin, inter- and intracontinental migration patterns, and dynamics of spread using the Bayesian framework.

## Methods

### Studied datasets

A detailed MLST database search performed in May 2023 revealed the presence of 69 sequences of *B. lusitaniae* strains, which were included in the initial alignment (https://pubmlst.org/bigsdb?db=pubmlst_borrelia_isolates&page=query). Sequences included in the study were aligned using the ClustalW algorithm implemented in MEGA X software package (Kumar et al., 2018).

### Phylogenetic analysis

Bayesian statistical approach was employed to infer the evolutionary relationship of analyzed sequences, using MrBayes 3.2.7 software (Ronquist et al., 2012). Detection of possible recombinants was performed using various models implemented in Recombination Detection Program v4 (RDP4) (Martin et al., 2015). To confirm the obtained results, corresponding phylogenetic subtrees were constructed according to the recombination positions identified in RDP4 program. Subtrees were built using maximum-likelihood (ML) algorithm, implemented in MEGA X software package.

The phylogenetic signal was evaluated by likelihood-mapping analysis implemented in the Tree-Puzzle program that estimates maximum-likelihood (ML) trees for all possible quartets of sequences and counts the frequency of trees according to their quality (Strimmer and von Haeseler, 1997). Temporal signal was examined by the root-to-tip regression approach implemented in the program TempEst (Rambaut et al., 2016). The best-fitting nucleotide substitution model was GTR+G+I, as selected by Akaike's information criterion (AICc) using jModelTest 3.06 (Posada, 2008).

### Phylogeographic analysis

The reconstruction of the ancestral discrete states was performed using Markov chain Monte Carlo (MCMC) sampling analysis over discrete sampling locations and a Bayesian stochastic search variable selection (BSSVS) approach, as implemented in BEAST v1.10.4 software (Lemey et al., 2009; Suchard et al., 2018). An uncorrelated lognormal relaxed lognormal molecular clock, together with a GMRF Bayesian Skyride tree prior was used as the best fitted for analyzed datasets. The MCMC chains were run for 4 × 10^7^ generations sampling every 40,000 generations with a burn-in of 10%. The convergence was assessed with Tracer v 1.7 based on the effective sampling size (ESS > 200) estimated for each parameter (http://beast.bio.ed.ac.uk/Tracer). Time-scaled maximum clade credibility (MCC) trees were visualized in FigTree v 1.4.4.

### Phylodynamic analysis

To reconstruct the evolutionary dynamics, using the birth-death skyline model (BDSKY), all previously defined clusters were analyzed. Analyses were performed in BEAST2 v 2.6.5 software package with literature-informed set of parameters (Bouckaert et al., 2019). Briefly, the effective reproductive number (Re) was set as a log-normal prior with a mean value (M) of 0.0 and a variance (S) of 1.25, with the number of dimensions set to four, five or 10 dimensions, as best suited for the particular clade. To visualize Re trends, the log output files of BEAST 2 were plotted using the “bdskytools” package in the R studio (https://github.com/cran).

## Results

In the present study, one dataset of 63 strains *B. lusitaniae* MLST sequences was subjected to phylogenetic analysis. The analyzed dataset was made of seven housekeeping loci (clpA, clpX, nifS, pepX, pyrG, recG, and rplB) originating from samples collected in eight European and two African countries between 1993 and 2023.

**Figure 1:**
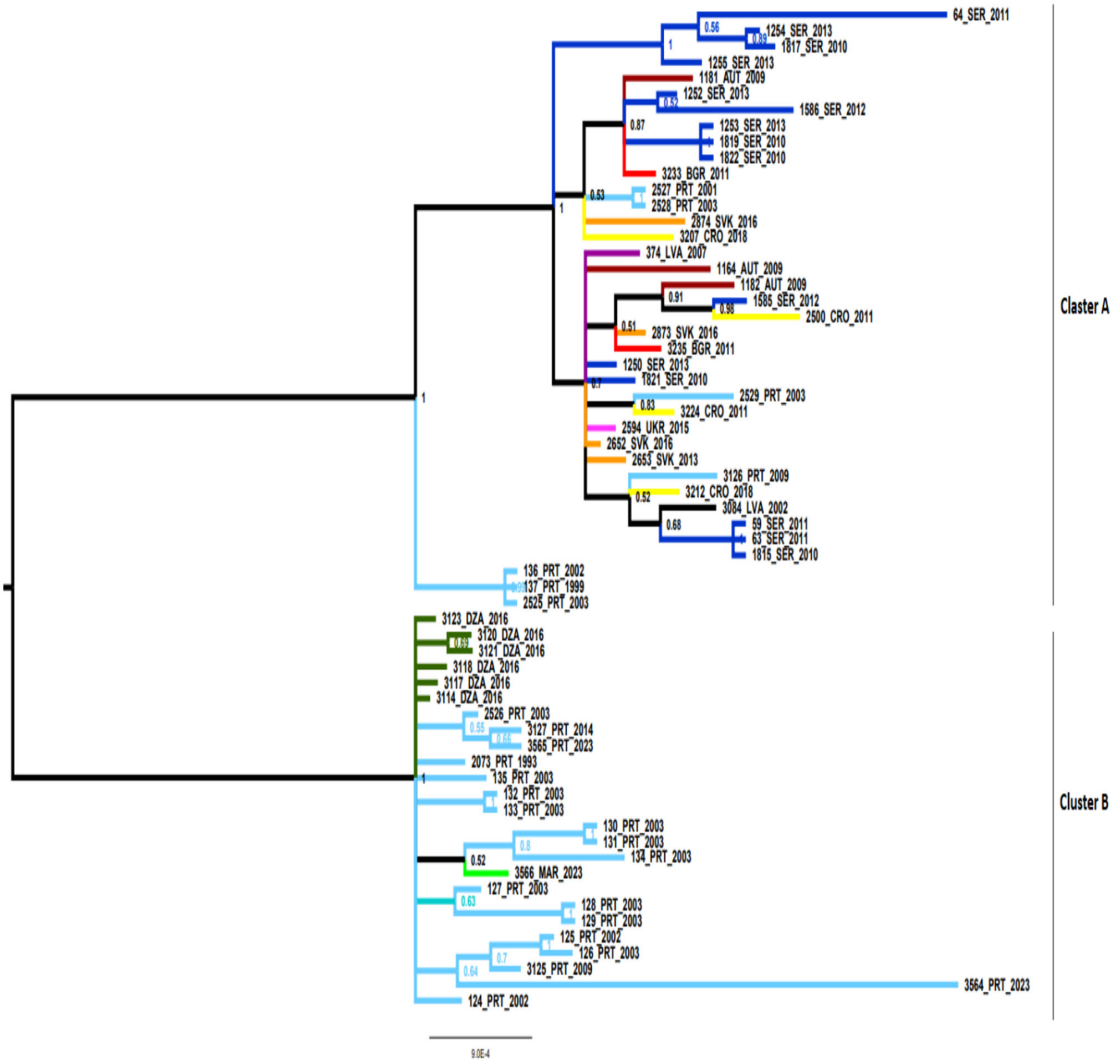
Bayesian phylogenetic tree of *B. lusitaniae* using 63 sequences of seven housekeeping *B. lusitaniae* loci. The numbers in bifurcations indicate posterior probabilities. Cluster A consisted of 38 European MLST isolates, while cluster B was composed 25 strains from Portugal, and North Africa (Algeria and Morocco). AUT, Austria; BGR, Bulgaria; CRO, Croatia; DZA, Algeria; LVA, Latvia; PRT, Portugal; SER, Serbia; SVK, Slovakia; UKR, Ukraine.

Bayesian phylogenetic analysis showed the presence of two well-defined clusters (cluster A and cluster B) (Figure 1). Cluster A represents European MLST isolates, including 38 European isolates, while cluster B was composed of 25 strains from Portugal, and North Africa (Algeria and Morocco).

To evaluate potential recombinants, the MLST sequence dataset was analyzed using the RDP4 program, which showed the presence of four potential recombination strains (isolates referred in the MLST database: PoHL1, PoTiBL37, PotiBmfP147, and GR24; labeled in the Figure 1 as 136, 137, 2,525, and 3,564 respectively. Three of these (PoHL1, PoTiBL37, and PotiBmfP147) showed the identical pattern of recombination in RDP4 program, corresponding to positions 1–568, 569–1,279, and 1,280–5,214 in the alignment, while GR24 recombinant strain had different pattern corresponding to positions 1–3,665 and 3,666–4,716.

Reconstructed phylogenetic subtrees were clearly in correlation with corresponding positions proposed by RDP4 analysis, with changing clustering of prospective recombinant sequences. According to these results three Portuguese strains may be considered as recombinant forms of sequences clustering with those from Central Europe and strains related to those from Portugal; the fourth strain may be considered as recombinant form of different strains from Portugal.

The likelihood mapping analysis showed that analyzed dataset contained sufficient genetic information for the phylogenetic analysis. The assessment of the temporal signal associated with the sequence alignment through root-to-tip regression analysis revealed a correlation coefficient of 0.204.

**Figure 2:**
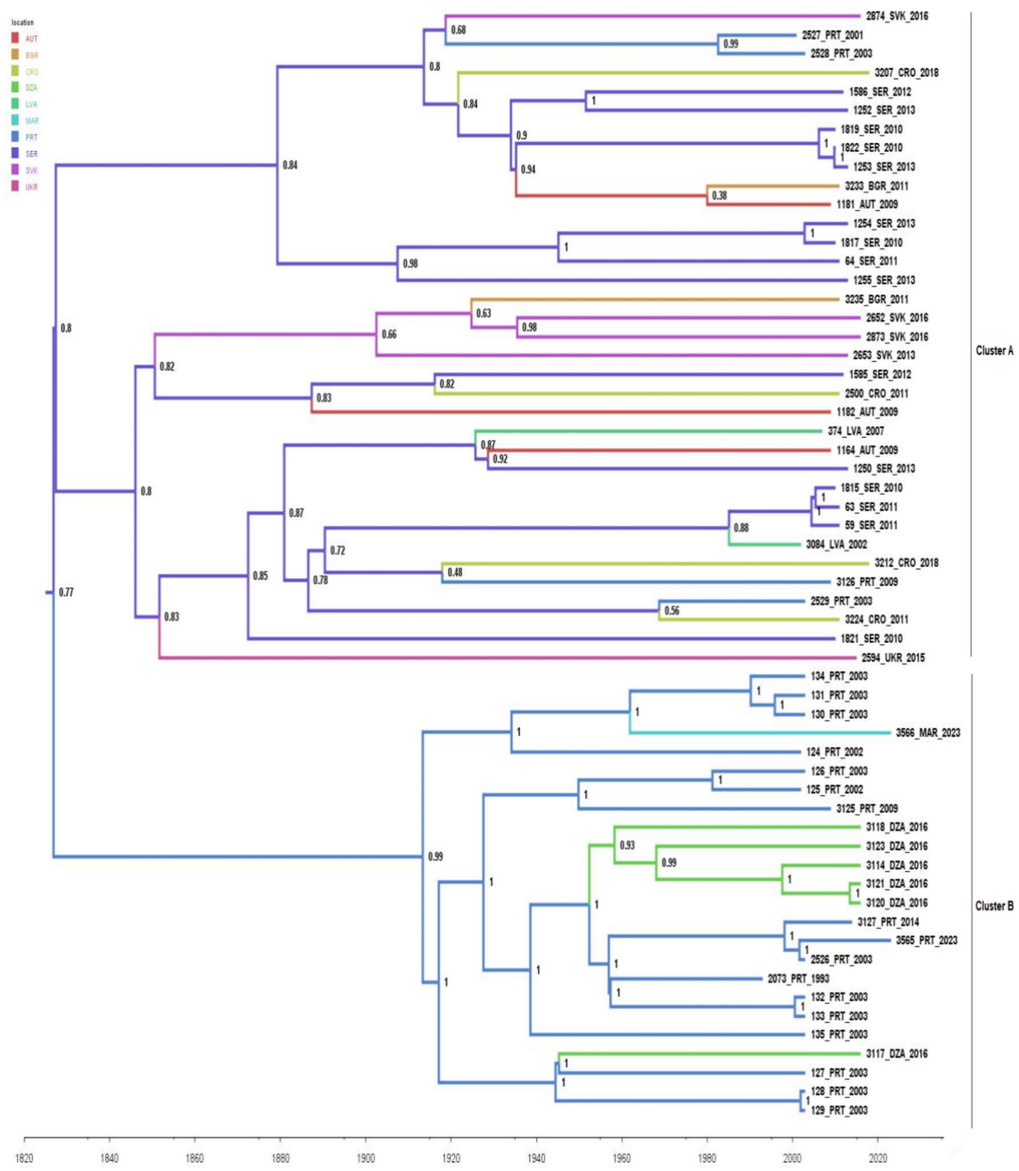
Phylogeographic analysis of 59 concatenated sequences of seven housekeeping *B. lusitaniae* loci performed in BEAST 1.10.4 software. Maximum clade credibility (MCC) tree was visualized in FigTree 1.4.4. The branches are colored based on the most probable location of the descendent nodes. The numbers on the internal nodes indicate significant posterior probabilities. AUT, Austria; BGR, Bulgaria; CRO, Croatia; DZA, Algeria; LVA, Latvia; PRT, Portugal; SER, Serbia; SVK, Slovakia; UKR, Ukraine.

To explore the migration routes of *B. lusitaniae* between continents and countries we employed a discrete-trait phylogeography analysis (Figure 2). Studied dataset encompassed 59 sequences collected between 1993 and 2023, from eight European and two African countries, since potential recombinant strains were excluded from further analysis. The obtained results showed that Serbia is likely the country of origin for *B. lusitaniae* in Europe and that transmission from Serbia to other European countries appears to be the main mechanism of spread in Europe. MCC tree shows a bifurcation at the root which leads to a large clade of European sequences, which splits close to the root, and a clade of sequences from Portugal and North Africa. Interestingly, almost all analyzed routes within large European clade representing a new introduction from Serbia to one of the European countries included in the analysis. Therefore, obtained results suggested multiple transmission of *B.lusitaniae* from Serbia to other European countries. Furthermore, introduction of *B. lusitaniae* to North Africa appears to have occurred via Portugal on at least two occasions.

**Figure 3:**
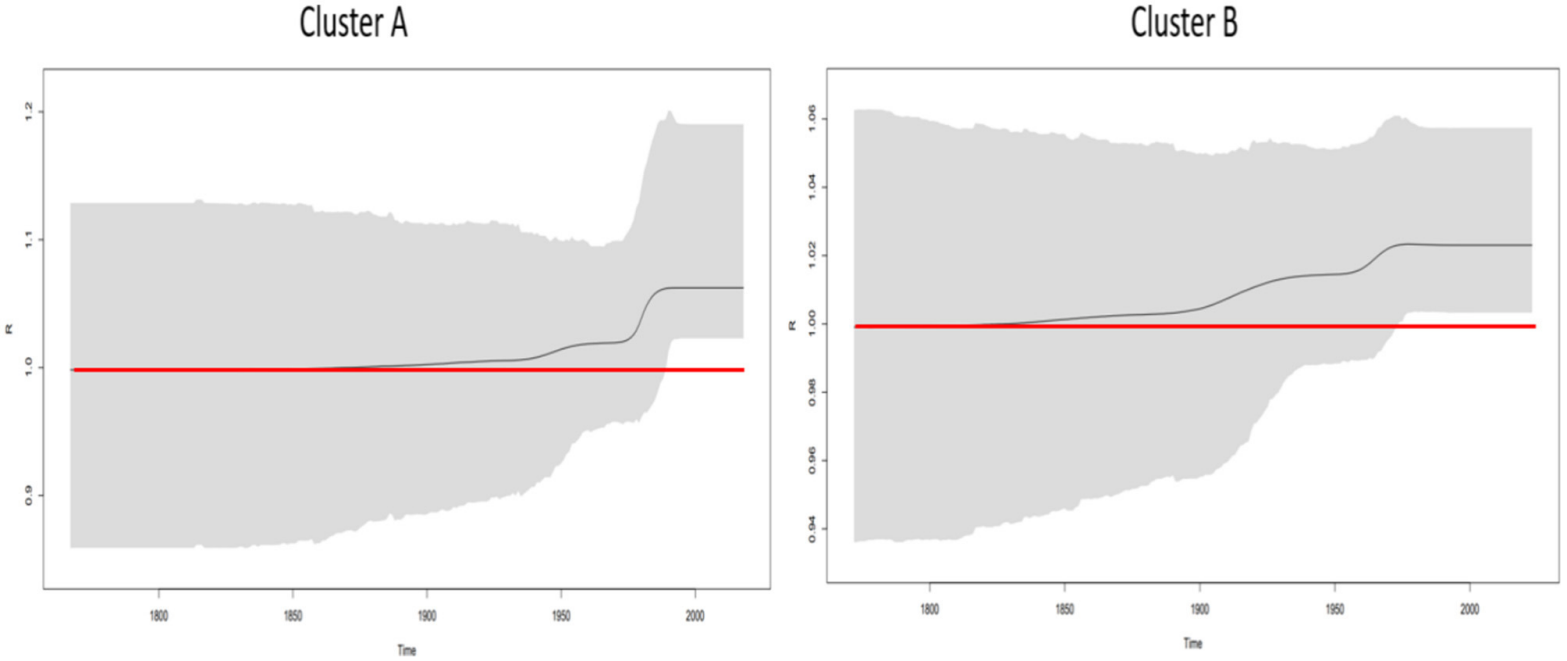
Birth-death skyline plot based on the seven housekeeping *B. lusitaniae* loci. BDSKY model, implemented in BEAST2 v2.6.5, was used. Cluster A consisted of 35 European MLST isolates, while cluster B was composed of 24 strains from Portugal, and North Africa (Algeria and Morocco). The red line delineates the cut-off value of Re=1. Shaded area represents 95% confidence intervals of Re estimates over time. X-axis represents time in years; Y-axis represents Re value.

The phylodynamics of *B. lusitaniae* was assessed by calculation of Re over time on two phylogenetic clusters (described above in the section phylogenetic analysis). The curve of mean Re values and 95%HPD using five intervals showed an increase starting from 1940 for European clade and from the beginning of 1900s for Portuguese and North African clade (Figure 3). Both investigated clades reached maximum soon after they became active and remained above one until the present time.

## Discussion

The evolution of zoonotic microparasites, maintained by vectors such as mosquitoes and ticks, is shaped by different environmental factors (Vitorino et al., 2008). The geographic distribution of *B. burgdorferi* s. l. species in Europe shows dynamics in spatial and temporal variations (Mannelli et al., 2012). Further, different *Borrelia* species show differences in pathogenicity and clinical manifestation of LB (Rudenko et al., 2009), thus, the knowledge of the geographic distribution and patterns of spreading of the pathogen is very important for understanding the ecology and epidemiology of the disease, allowing adequate risk assessment. Considering that *B. lusitaniae* was isolated from the skin and blood of patients with chronic skin lesions (Collares-Pereira et al., 2004; Tomanović et al., 2023), it is reasonable to suggest that studied *Borrelia* species is potentially pathogenic for humans.

Here, we present the phylogenetically based investigation of migration patterns of *B. lusitaniae*, as a pathogen potentially responsible for clinical manifestations of LB. To our best knowledge, this is the first study that explores the phylogeographic spread and phylodynamics of *B. lusitaniae*, as a pathogen potentially responsible for clinical manifestations of LB.

Bayesian phylogenetic tree was constructed based on 63 sequences of seven *B. lusitaniae* concatenated housekeeping genes. The results of analysis pointed toward the spatial differentiation, based on geographic origin and pattern of migratory flyways, showing separation between North African and European isolates. The existence of this type of geographically related clustering reflects the fact that strains of *B. lusitaniae* can be recognized either as Mediterranean or European type. The only exception has been noticed in Portuguese isolates since they have been presented in both subclades (subclade A and subclade B).

Similar findings were also observed by employing the Bayesian phylogeographic approach to reconstruct the dispersal history of 59 *B. lusitaniae* “MLST” sequences. The obtained results suggested that the Serbia was the country origin for *B. lusitaniae*. This result of root-to-tip analysis implied that the molecular clock analysis may underestimate the real age of the deepest splits of the phylogeny and therefore we did not discuss about the age of *B. lusitaniae* origin. Our phylogeographic analysis suggested multiple transmission events of this spirochete from Serbia to other European countries. Introduction of *B*. *lusitaniae* into Portugal led to the further local spread of this pathogen but also into North African countries.

The genetic difference between two geographically close Portuguese populations could be the result of some geographical barriers (Norte et al., 2021). Specifically, in this case the river Tagus may represents geographic obstacle for the local spread of *B. lusitaniae* in Portugal, the river Tagus fell into cluster A while samples from south of the river Tagus fell into cluster B. The presence of two genetically different populations of *B. lusitaniae* in Portugal can be also explained by the occurrence of additional vector species from genus *Ixodes* in this region. In 2014, a new *Ixodes* species was described and named *Ixodes inopinatus* (Estrada-Peña et al., 2014). This new species can be found in the western Mediterranean: Spain, Portugal, Morocco, Algeria, and Tunisia. In Spain, its northern limit is the province of Guadalajara (Petney et al., 2015). The former study, proposed by Norte et al. (2021), hypothesized that the existence of two phylogenetically different *B. lusitaniae* populations may be the consequence of association with different vector species. However, the obtained results did not explicitly support the proposed hypothesis. Having all this in mind it is not possible unambiguously to point out the role of *I. inopinatus* in the population division of *B. lusitaniae* in Portugal.

Lastly, lizards, as hosts of *B. lusitanie* also play important role in geographic distribution of this pathogen. *B. lusitanie* has been associated with various species of lizards; Algerian lizard (*P. algirus*) in Spain and North Africa, green lizards (*L*. *viridis*) in Slovakia, sand lizards (*L. agilis*) in Germany and common wall lizards (*Podarcis muralis*) in Italy (Norte et al., 2021). Previous findings underlined the fact that Mediterranean lizard species within the family *Lacertidae*, previously described as reservoirs of *B*. *lusitaniae*, are highly parapatric (Vitorino et al., 2008). Therefore, our results are in accordance with previous studies (Vitorino et al., 2008; Norte et al., 2021) and highlight the significant role of lizards in the maintenance of local cycles of *B. lusitaniae* throughout Portugal. The focal distribution of this spirochete in Central and Northern Europe can also be explained as the consequence of the narrow ecological niche of lizards. Specifically, the population structure of pathogens that use hosts with smaller migration ranges, such as rodents and lizards, is more pronounced. Consequently, the limited distribution of different lizard species associated with *B*. *lusitaniae* restricted to certain countries/geographic areas is likely to have implications on *B. lusitaniae* evolution and epidemiology. Moreover, results obtained in the study of Grego and colleagues, regarding phylogenetic analysis of *B. lusitaniae* OspA gene sequences, also suggested the existence of two different *B. lusitaniae* strains, circulating in Europe and North Africa (Grego et al., 2007). However, the existence of potential recombinants only in Portuguese strains may suggest that mixing of two *B. lusitaniae* populations may still occur. Specifically, one Portuguese strain (GR24), that could be considered to be recombinant of different strains from Portugal, may evidence that the process of mixing two different *B. lusitaniae* populations is possible. Similar to this, other three recombinant strains (PoHL1, PoTiBL37, and PotiBmfP147) most possible represent the result of intermixing process between Mediterranean and European *B. lusitaniae* populations.

Two phylogenetically distinguished populations of *B. lusitaniae* (Mediterranean and European) are the consequence of several refuges created after the last period of glaciations (Schmitt and Varga, 2012). However, climate changes had a significant impact on the expansion of *Borrelia* species since most of life cycle of *I. ricinus* occurs outside the hosts and therefore it's directly influenced by temperature and humidity changes at the microclimatic level (Mannelli et al., 2012). The detailed phylogenetic analysis of recombinant strains presented in this study, indicates that the gene flow may exist. Given that there are some major geographic obstacles (mountains and rivers) between Central Europe and Portugal, intermixing of two populations, is possible via the major migratory bird flyways that could play an important role in the intercontinental dispersal patterns of *B. lusitaniae*, acting as long-distance dispersal vehicles. Birds may also be responsible for intermixing of *B. lusitaniae* within Mediterranean region. Results of our study implied that introduction of *B. lusitaniae* to North Africa took place via Portugal at least two times. In this case, Mediterranean Sea represents geographical constraint that only birds can cross. Previous study, aimed to analyze the evolutionary history of B. *burgdorferi s.s*. in North America, also reported the evidence of long-distance migration events between different geographic regions, possibly due to long-distance, bird-mediated dispersal. This gene flow was explained by the recombination events of short genomic fragments and shuffling of entire plasmids, detected in this study (Walter et al., 2017). However, the dynamics of all these processes still need to be studied, since this type of analysis requires a larger number of sequences deposited in the database.

Portugal as a European country geographically close to North Africa can be labeled as the bridge which most likely facilitates rapid long-distance dispersal between Europe and North Africa. This agrees with our results suggesting at least two introduction events of *B. lusitaniae* from Portugal to North African countries (Algiers and Morocco). Furthermore, genetic similarity between most Portuguese and all North African strains, reported in the present study, also supported the statement about the central importance of Portugal for the intercontinental spread of *B. lusitaniae*. The question that remains to be answered is whether the birds directly “bring” *B. lusitaniae* from Portugal to North Africa, or via Spain. The presence of *B*. *lusitaniae* in Spain has already been reported (Díaz et al., 2017), but there are no available Spanish “MLST” sequences in the database which can be included in the present study. However, since the Spanish mainland is the closest European country to North Africa, it is reasonable to hypothesize that birds may cross Gibraltar from Spain to Africa and ingress *B*. *lusitaniae* in North African countries.

Described phylogenetic structure of *B. lusitaniae* indicates that the major flyways of migratory birds could play an important role in the intercontinental dispersal patterns of *B. lusitaniae*. Importance of migratory birds, related to the spread and maintenance of different tick-borne pathogens has already been documented in previous studies (Alekseev et al., 2001; Poupon et al., 2006; Hasle et al., 2011; Socolovschi et al., 2012). Birds have the potential to spread ticks and tick-borne pathogens, by easily crossing the geographic barriers, on long distances in a relatively short time (Hasle, 2013). The study conducted in Switzerland suggested that birds seem to be reservoir hosts for *B. lusitaniae*, since only larvae were feeding on birds (Poupon et al., 2006). The prevalence of *B. lusitaniae* in Europe, excluding Southwest Europe, was very low and restricted only to some countries until 15 years ago. However, in 2008 Milutinović et al. published a paper about the prevalence of tick-borne bacterial pathogens in *I. ricinus* ticks, collected from vegetation in 2001, 2003, and 2004 in Serbia. The most common *B. burgdorferi s.l*. species was *B. lusitaniae*. In the following years, *B. lusitaniae* was found to be one of the most dominant *Borrelia* species on the territory of Serbia (Potkonjak et al., 2016; Cakić et al., 2019). The results of all these studies unambiguously implied the importance of Serbia for the spatial spread of *B. lusitaniae* throughout Central and Southeast Europe suggested in this study. However, the prevalence of *B. lusitaniae* in ticks collected from lizards in Serbia is still unknown. Most of the information on *B. lusitaniae* presence and distribution in Serbia were derived from questing ticks, except ticks from dogs and golden jackals (Potkonjak et al., 2016; Sukara et al., 2018), and therefore it is difficult to explain in which way studied pathogen spread within and beyond the Serbian borders. In addition, *B. lusitaniae* was found in spleen samples of red foxes (Sukara et al., 2019).

Bird ringing is the scientific method developed to obtain demographic data about the migration routes of birds. Using data obtained from EURING (https://euring.org/) we analyzed the migratory routes of bird flights between Serbia and other European countries (Slovakia, Ukraine, Croatia) which have already reported the presence of *B. lusitaniae* (Majláthová et al., 2006; Taragelová et al., 2016; Weiner et al., 2018; Norte et al., 2021). Analyzed routes confirmed that those birds within families *Sylviidae, Muscicapidae, Turdidae*, and *Acrocephaliidae* ringed in Serbia in different time points were found in the mentioned countries. These findings suggest that birds play an important role in maintaining *B. lusitaniae* in endemic areas but also in its spread between countries by traveling carrying infected ticks. In addition to this possibility, birds as potential hosts of *B. lusitaniae* can be infected by this pathogen and successfully transmit it to uninfected ticks during next feeding. The last known mechanism that enables the transmission of *Borreliae* species is the tick co-feeding on birds with ticks infected with one species of *Borrelia* (Hasle, 2013).

To explore the temporal trend of the *B. lusitaniae* distribution in Europe and North Africa, we analyzed the phylodynamics of the two previously defined clades, using a birth-death skyline model. For clades A and B of studied dataset, an increase in activity was seen from 1900. Although our results suggested the expansion of *B. lusitaniae* from the beginning of the 20^th^ century, intensive spread has been noticed in the last 15 years. One of the possible reasons for this deviation is the fact that the number of potential hosts species associated with *B. lusitaniae* has increased over time (Poupon et al., 2006; de Carvalho et al., 2010; Norte et al., 2015; Sukara et al., 2018, 2019). Global warming has also imposed an immense threat to global biodiversity, including reptiles as ectothermic animals. A former study, conducted on reptiles in Spain, revealed statistically significant shift in their distribution northward in response to climate changes (Moreno-Rueda et al., 2012). Changes in the geographic distribution of hosts directly affect the change in the ecological niche of vectors, in this case ticks, and therefore on pathogens transmitted by ticks.

Even though estimated Re implies the potential for the spread of *B. lusitaniae*, this pathogen is not transmissible within human populations and cannot consequently cause substantial epidemics (Stanek and Strle, 2018). Therefore, outbreak size mostly depends on the number of introductions from animal hosts and ticks as vectors.

The present study represents the first detailed phylogenetic characterization of *B. lusitaniae*, based on the available MLST sequences. In-depth phylogenetic analysis revealed that *B. lusitaniae* originated most probably in Serbia and spread further on throughout Europe and to North Africa. Estimated Re value for analyzed clades was >1, suggesting the potential for further spread. The key aspects of this study were to sum up and to analyse genetic data related to *B. lusitaniae* as promising areas for further ecological studies. The potential limitation of this most comprehensive investigation so far is the lack of the overall number of MLST sequences from all countries that have previously reported the presence of this pathogen. Furthermore, the lack of whole genome sequences in the database, which can provide insight into the deepest phylogeny, also represents the potential limitation and therefore the present study underscored the relevance of the next-generation sequencing (NGS) approach for this type of analysis.

## Data availability statement

Publicly available datasets were analyzed in this study. This data can be found here: https://pubmlst.org/bigsdb?db=pubmlst_borrelia_isolates&page=job&id=BIGSdb_3875253_7213740707_09690.

## Author contributions

VC: Conceptualization, Investigation, Methodology, Visualization, Writing – original draft, Writing – review & editing. GV: Writing – review & editing. DS: Methodology, Writing – review & editing. DM: Writing – review & editing. RS: Writing – review & editing. ST: Conceptualization, Funding acquisition, Validation, Writing – review & editing.
