# Supplementary material for: Evolutionary dynamics and geographical dispersal of Borrelia lusitaniae
Source: Front Microbiol. 2024 Feb 6;15:1330914. doi: 10.3389/fmicb.2024.1330914 (PMC10877945; doi:10.3389/fmicb.2024.1330914)
Supplement: Supplementary file 1 [file Data_Sheet_1.PDF]

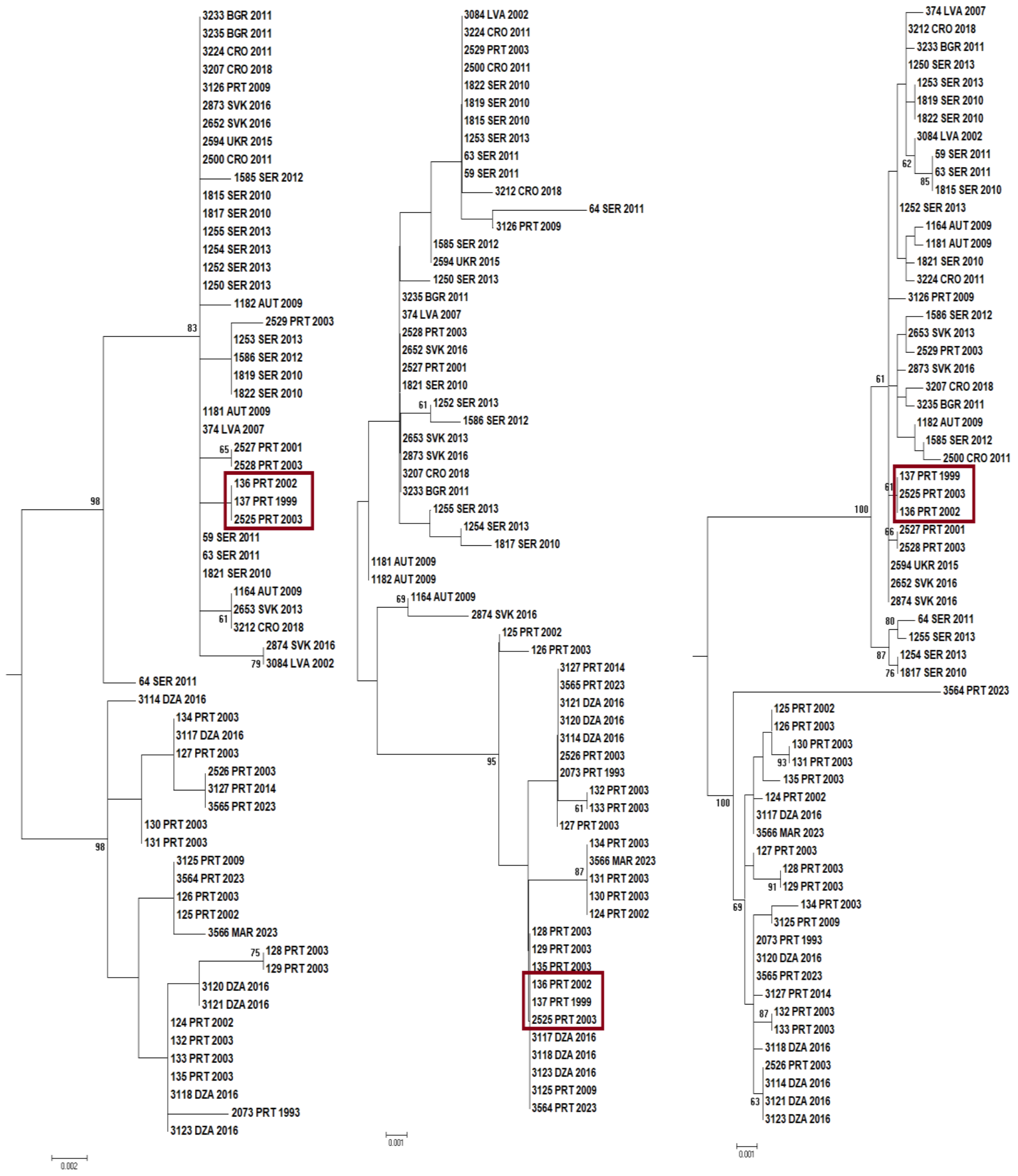

Figure S1. Reconstructed phylogenetic subtrees based on the positions identified in the RDP4 analysis. Subtrees were built using maximum-likelihood (ML) algorithm, implemented in MEGA X software package. Complete alignment of 4215 nt was split in three subalignments (1-568 nt, 569-1279 nt and 1280-5214 nt) to analyze recombination pattern in three sequences (136, 137 and 2525) belong to cluster A. The numbers in bifurcations indicate bootstrap values.

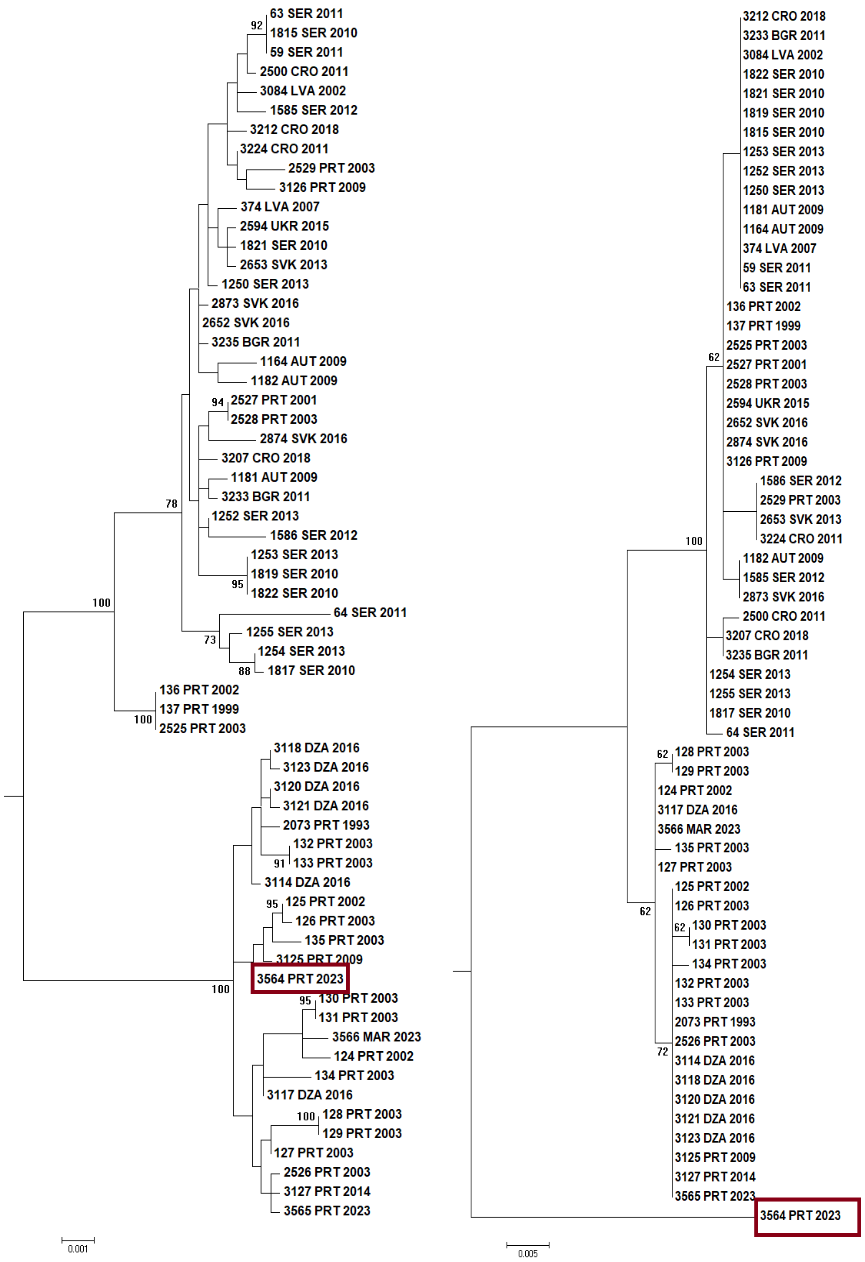

Figure S2. Reconstructed phylogenetic subtrees based on the positions identified in the RDP4 analysis. Subtrees were built using maximum-likelihood (ML) algorithm, implemented in MEGA X software package. Complete alignment of 4215 nt was split in in two subalignments (1–3665 nt and 3666–4176 nt) to analyze the recombination pattern in sequence (3564) belongs to cluster B. The numbers in bifurcations indicate bootstrap values.
